# Protective Effect of 3,4-Methylenedioxyphenol (Sesamol) on Stress-Related Mucosal Disease in Rats

**DOI:** 10.1155/2013/481827

**Published:** 2013-07-25

**Authors:** Dur-Zong Hsu, Yi-Wei Chen, Pei-Yi Chu, Srinivasan Periasamy, Ming-Yie Liu

**Affiliations:** ^1^Department of Environmental and Occupational Health, National Cheng Kung University Medical College, Tainan 70428, Taiwan; ^2^Sustainable Environment Research Center, National Cheng Kung University, Tainan 70101, Taiwan

## Abstract

Stress-related mucosal disease (SRMD) causes considerable morbidity and mortality in critically ill patients. 3,4-Methylenedioxyphenol (sesamol) has been reported to have potent antioxidative and anti-inflammatory properties. The aim of this study was to investigate the effect of sesamol on water immersion restraint- (WIR-) induced SRMD in rats. Rat gastric ulcer and hemorrhage were induced by WIR. Rats were pretreated orally with various doses of sesamol (0.1, 0.3, and 1 mg/kg, resp.) 30 min before WIR. Gastric mucosal ulceration, hemoglobin, lipid peroxidation, mucus secretion, proinflammatory cytokines, and nuclear factor (NF)-**κ**B levels were determined 4 h after WIR. In addition, the infiltration of neutrophil and macrophage into gastric mucosa was also determined after WIR. Water immersion restraint increased gastric mucosal ulcer and hemorrhage, tumor necrosis factor (TNF)-**α**, interleukin (IL)-1**β**, and IL-6 levels but failed to affect mucosal lipid peroxidation and mucus secretion compared with non-WIR. Sesamol significantly decreased gastric ulceration and hemorrhage and inhibited mucosal TNF-**α**, IL-1**β**, and IL-6 production and NF-**κ**B activity in WIR-treated rats. In addition, increased myeloperoxidase and CD68 levels in gastric mucosa were found in WIR-treated rats compared to non-WIR rats. Sesamol did not affect myeloperoxidase but decreased CD68 levels in mucosa in WIR-treated rats. Sesamol may protect against SRMD by inhibiting gastric mucosal proinflammatory cytokines in rats.

## 1. Introduction

Stress-related mucosal disease (SRMD) (i.e., gastric mucosal damage, ulceration, and bleeding) causes considerable morbidity and mortality in critically ill patients [1]. Patients with stress ulceration and bleeding have a longer ICU stay and a higher mortality than patients who do not have stress ulcers [1]. Moreover, the mortality rate from stress-related mucosal bleeding is nearly 50% [2]. Antacids such as histamine H_2_ receptor antagonist and proton pump inhibitor have been used to treat patients with stress ulcer; however, they have been suggested to be associated with the incidence of nosocomial pneumonia and the inhibition of immune function [3–5].

Water immersion restraint (WIR), a model that mimics the clinical acute gastric hemorrhage and ulceration, has been widely accepted for studying stress-related gastric mucosal injury [6, 7]. Increasing gastric lipid peroxidation [8] and oxidative stress [9] and decreasing mucus production [10] have been suggested to be involved in the pathogenesis of WIR-induced gastric hemorrhage and ulceration. Further, gastric mucosal inflammation is also important in stress-associated gastric mucosal damage [11, 12]. 

During gastric mucosal inflammation, activated phagocytes such as macrophages and neutrophils infiltrate into inflammatory organ. Activating nuclear factor (NF)-*κ*B, a transcriptional factor which regulates the transcription of inflammation-related gene, leads to a release of proinflammatory cytokines [13], such as tumor necrosis factor (TNF)-*α*, interleukin (IL)-1*β*, and IL-6. Pro-inflammatory cytokines disrupt mucosal protective mechanisms and therefore damage the gastric mucosa [14]. 

 Sesamol (3,4-methylenedioxyphenol), a lignan in sesame oil, has potent antioxidative and anti-inflammatory properties [15]. Sesamol has protective effect in nonsteroidal anti-inflammatory drugs-induced gastric ulcer [16]. However, the protective effect of sesamol in stress-associated ulcer has never been investigated. The aim of the present study was to examine the protective effect of sesamol on stress-induced gastric mucosal hemorrhage and ulceration in rats.

## 2. Methods

### 2.1. Materials

Sesamol was purchased from Sigma (St. Louis, MO, USA). Sesamol was prepared freshly (1 : 1 w/v in water).

### 2.2. Animals

Male SPF Wistar rats weighing 300 to 350 g (8 weeks old) were purchased from and housed in our institution's Laboratory Animal Center. Rats were kept in a room with 12 h light/dark cycle and with central air condition (25°C, 70% humidity). They were allowed free access to tap water and a pelleted rodent diet (Richmond Standard; PMI Feeds, Inc., St. Louis, MO, USA). The animal care and experimental protocols were in accord with nationally approved guideline. 

### 2.3. WIR-Induced Stress-Related Gastric Mucosal Injury in Rats

Stress-related gastric mucosal injury was induced by WIR. After being fasted overnight, rats were restrained on plastic boards with elastic cord. Then, rats were immersed up to the depth of xiphoid level in a 25°C water bath for 4 h [17].

### 2.4. Experimental Design

#### 2.4.1. Experimental Design I

Rats were divided into five groups of five: Group I, non-WIR (normal, vehicle: water) group; Group II, WIR (control, vehicle: water) group rats were subjected to WIR; Groups III–V, sesamol-plus-WIR group rats were orally given sesamol (0.1, 0.3, and 1 mg/kg, resp.) 30 min before WIR. Gastric ulcer index, gastric hemorrhage, gastric tissue lipid peroxidation (LPO), mucus production, mucosal cytokine levels, mucosal NF-*κ*B activation, and mucosal myeloperoxidase (MPO) activity were assessed after WIR.

#### 2.4.2. Experimental Design II

Rats were divided into three groups of five: Group I, non-WIR (normal) group; Group II, WIR (control) group rats were subjected to WIR; Group III, sesamol-plus-WIR rats were given sesamol (1 mg/kg; p.o.) 30 min before WIR. Gastric mucosal morphologic and histological changes were assessed after WIR. In addition, CD68 stain in gastric mucosa was also conducted. 

### 2.5. Assessing Gastric Mucosal Ulceration

Stress-related gastric mucosal injury was assessed by determining the ulcerated area and by histological examination. To determine the ulcerated area, we opened the stomach along the greater curvature, and the length and width of the ulcers on the gastric mucosa were measured with a planimeter (1 × 1 mm) under a dissecting microscope. The surface area of each ulceration was calculated by assuming that it was an ellipse (length × width × *π*/4), and the total area was summated and expressed in square millimeters. For the histological examination, briefly, stomach tissue was fixed in 4% formaldehyde buffered with a phosphate solution (0.1 M (pH 7.4)) at room temperature. Tissue fragments were washed in phosphate buffer, dehydrated in graded concentrations of ethanol, and then embedded in paraffin. From each tissue, 4 *μ*m thin sections were cut, stained with hematoxylin and eosin, and examined under a light microscope [16].

### 2.6. Measuring Gastric Hemorrhage

To measure gastric hemorrhage, stomachs were lavaged with 4 mL of saline. After centrifuging (2500 rpm, 10 min), the volume of supernatant was measured and the Hb contents of the samples were measured spectrophotometrically (376 nm) [18]. The volume multiplied the hemoglobin concentration to get the final content and the unit showed as mg hemoglobin/stomach. 

### 2.7. Measuring Gastric LPO

Mucosal tissue was homogenized in Tris-HCl (20 mM, pH 7.4). The homogenate was centrifuged at 2500 ×g at 4°C for 10 min. The supernatant was taken for LPO measurement. The measurement was using a commercial assay kit (MDA-586 assay kit; OXIS international, Inc., USA). The absorbance at 586 nm was detected by the spectrophotometer (DU 640B; Beckman, Fullerton, CA, USA) [16].

### 2.8. Assessing Mucus Secretion

Mucus secretion was assessed by measuring mucosal hexosamine levels. Gastric mucosal mucin was extracted with Triton X-100 and hydrolyzed by 4 N HCl. Hexosamine obtained form hydrolysed mucin reacted with acetylacetone and Ehrlich's reagent, and the absorbance at 537 nm was detected [19].

### 2.9. Measuring Gastric Mucosal TNF-*α*, IL-1*β*, and IL-6

TNF-*α*, IL-1*β*, and IL-6 were measured using commercial enzyme-linked immunosorbent assay kits (DuoSet; R&D System, Minneapolis, MN, USA). Briefly, a 96-well immunoassay plate was coated with 100 *μ*L/well capture-antibody overnight at room temperature. After blocking step, samples homogenizing with water and standards were loading into each well (100 *μ*L/well) and were incubated at room temperature for 2 h. One hundred *μ*L of biotinylated rabbit anti-rat TNF-*α*, IL-1*β*, and IL-6 antibodies was then added and incubated at room temperature for 2 h. After antibody capturing, streptavidin-conjugated horseradish peroxidase was added and was incubated at room temperature for 20 min. The peroxidase reaction was initiated by adding 100 *μ*L of 3,3′,5,5′-tetramethylbenzidine/H_2_O_2_ (R&D systems) for 30 min and then was stopped by adding 50 *μ*L of 0.5 M H_2_SO_4_. The absorbance was measured at 450 nm using an enzyme-linked immunosorbent reader [20].

### 2.10. Measuring Mucosal NF-*κ*B Activation

Nuclear protein extraction kit (Sigma, Inc, St. Louis, MO, USA) was used to isolate nuclear protein in gastric mucosa. NF-*κ*B was detected by the chemiluminescent NF-*κ*B assay kits (Thermo Scientific, Inc, Rockford, IL, USA). In brief, nuclear protein was loaded to the 96-well plate and bound to the biotin-Duplex. After incubation, the primary antibody and the secondary antibody-HRP were added. And then, chemiluminescent substrate was added, and the luminescence was detected with Fluoroskan Ascent FL (Thermo Fisher Scientific Inc, Waltham, MA, USA).

### 2.11. Measuring MPO Activity in Gastric Tissue

Gastric mucosal MPO, a marker enzyme of neutrophil infiltration, was detected. Tissue samples were homogenized in 20 mM phosphate buffer (pH 7.4) and centrifuged (13,000 rpm, 10 min, 4°C), and the resulting pellet was resuspended in 50 mM phosphate buffer (pH 6.0) containing 0.5% wt/vol hexadecyltrimethylammonium bromide (Sigma). The suspension was subjected to four cycles of freezing and thawing and further disrupted by sonication (40 s). The sample was then centrifuged (13,000 rpm, 5 min, 4°C), and the supernatant was mixed with equal volume of 1-component TMB Peroxidase Substrate (Sigma), incubated for a 1 min, and then terminated by equal volume of 2 N H_2_SO_4_. The absorbance was measured at 405 nm and corrected for the calculated tissue weight [21].

### 2.12. CD68 Stain in Gastric Mucosa

To assess macrophage recruitment in gastric mucosa, we stain CD68 (macrophage marker) in gastric mucosa. Tissue sections were deparaffinized, rehydrated, and then incubated with CD68 Ab-3 (clone KP1) antibody (1 : 200) (Thermo Fisher Scientific Inc., Fremont, CA, USA) for 30 min at room temperature and developed (Ultra Vision Detection System Anti-Polyvalent, Alk-Phos/BCIP/NBT (Ready-To-Use) Kit; Thermo Fisher Scientific). The sections were counterstained with Nuclear Fast red, cleared, and mounted using 3H-diethylphenylxanthine (DPX). CD68 positive cells were identified as blue color (100x).

### 2.13. Statistical Analysis

Data are means ± standard deviation (SD). One-way ANOVA and then the Tukey Honestly Significant Difference method were used to make pairwise comparisons between the treatments. Statistical significance was set at *P* < 0.05.

## 3. Results

### 3.1. Effect of Sesamol on WIR-Induced Gastric Mucosal Damage

**Figure 1:**
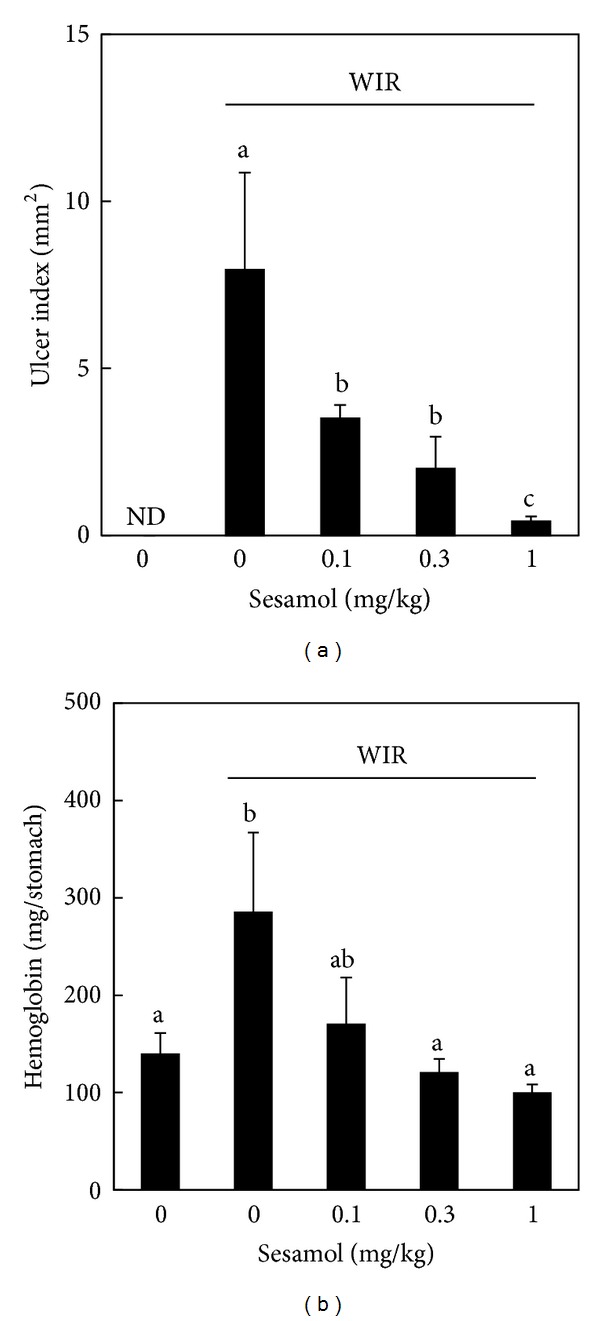
Effect of sesamol on water immersion restraint- (WIR-) induced gastric mucosal damage. Rats were divided into five groups of five: Group I, non-WIR (normal) group; Group II, WIR (control) group rats were subjected to WIR; and Groups III–V, sesamol-plus-WIR group rats were orally given sesamol (0.1, 0.3, and 1 mg/kg, resp.) 30 min before WIR. Gastric ulcer index (a) and hemorrhage (b) were assessed after WIR. Data are means ± SD. Significant differences between measurement traits were analyzed using one-way ANOVA. Different letters between groups indicate statistically significant differences (*P* < 0.05).

To examine the protective effect of sesamol on stress-related mucosal injury, gastric mucosal ulceration and hemorrhage as well as histological changes were assessed. WIR (control) group increased ulcer index and luminal hemoglobin levels compared with non-WIR group, while sesamol (sesamol-plus-WIR group) decreased both indicators compared to WIR group rats (Figures 1(a) and 1(b)).

### 3.2. Effects of Sesamol on Morphological and Histological Changes in WIR-Treated Rats

**Figure 2:**
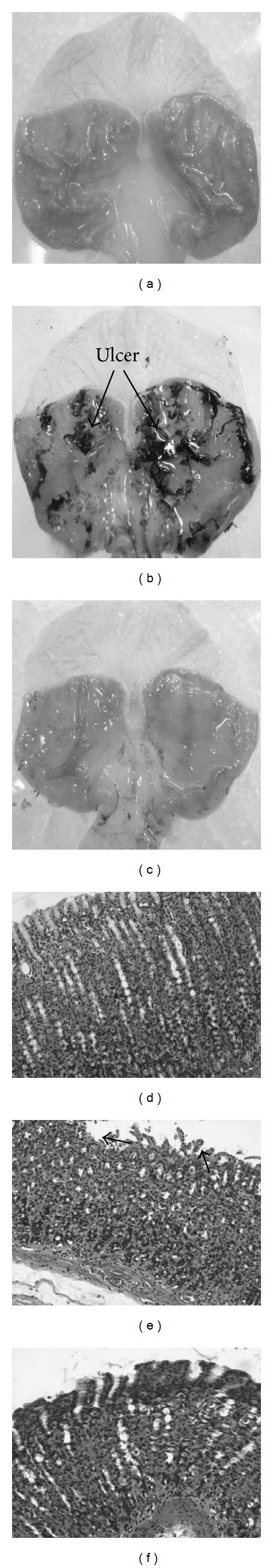
Effects of sesamol on morphological and histological changes in water immersion restraint- (WIR-) treated rats. Rats were divided into three groups: Group I, non-WIR (normal) group ((a), (d)); Group II, WIR (control) group rats were subjected to WIR ((b), (e)); and Group III, sesamol-plus-WIR group rats were orally given sesamol (1 mg/kg) 30 min before WIR ((c), (f)). Morphological ((a)–(c)) and histological ((d), (e)) changes were assessed after WIR. Non-WIR ((a), (d)) and WIR-plus-sesamol (c, f) groups appeared normal in both morphological and histological examinations. WIR group showed a large hemorrhagic ulcerated area (b) and a loss of the histological structure and a deep alteration of glandular epithelium serious lesions on mucosa (e) (hematoxylin-eosin, original magnification ×100). Arrow indicates disruption of surface epithelial cells of mucosa.

We used morphological and histological studies to further confirm the protective effect of sesamol on stress-induced gastric mucosal injury. Gastric mucosa from the non-WIR group (Figures 2(a) and 2(d)) appeared normal in both morphological and histological examinations. Gastric mucosa from the WIR (control) group showed a large hemorrhagic ulcerated area (Figure 2(b)). WIR induced mucosal congestion and disruption of surface epithelial cells with severe inflammatory cell infiltration at the base of mucosa, and necrosis was observed scattered in both intravascular and extravascular spaces throughout the mucosa (Figure 2(e)). In the sesamol-plus-WIR group, there was only a small ulcerated area in the mucosa (Figure 2(c)) which showed significant reduction in the disruption of surface epithelial cells with severe inflammatory cell infiltration and necrosis in the mucosa (Figure 2(f)).

### 3.3. Effects of Sesamol on Mucosal LPO and Mucus Secretion in WIR-Treated Rats

**Figure 3:**
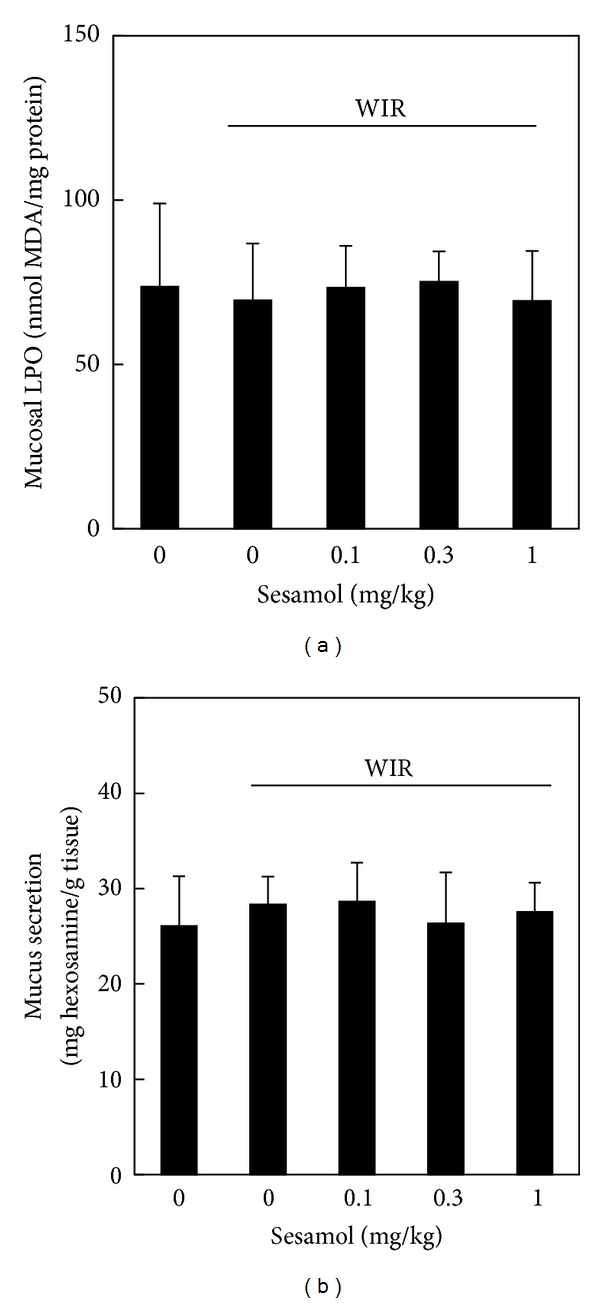
Effects of sesamol on mucosal lipid peroxidation (LPO) and mucus secretion in water immersion restraint- (WIR-) treated rats. Rats were divided into five groups of five: Group I, non-WIR (normal) group; Group II, WIR (control) group rats were subjected to WIR; and Groups III–V, sesamol-plus-WIR group rats were orally given sesamol (0.1, 0.3, and 1 mg/kg, resp.) 30 min before WIR. Mucosal malondialdehyde (MDA) and mucus hexosamine levels were determined after WIR.

To examine the involvement of oxidative stress and mucus secretion in sesamol-exerted gastric mucosal protection against stress, mucosal LPO level and mucus secretion were assessed. However, neither WIR (control) group nor sesamol-plus-WIR group showed difference in mucosal LPO (Figure 3(a)) and mucus production (Figure 3(b)) compared to non-WIR group.

### 3.4. Effects of Sesamol on Mucosal Proinflammatory Cytokines Production in WIR-Treated Rats

**Figure 4:**
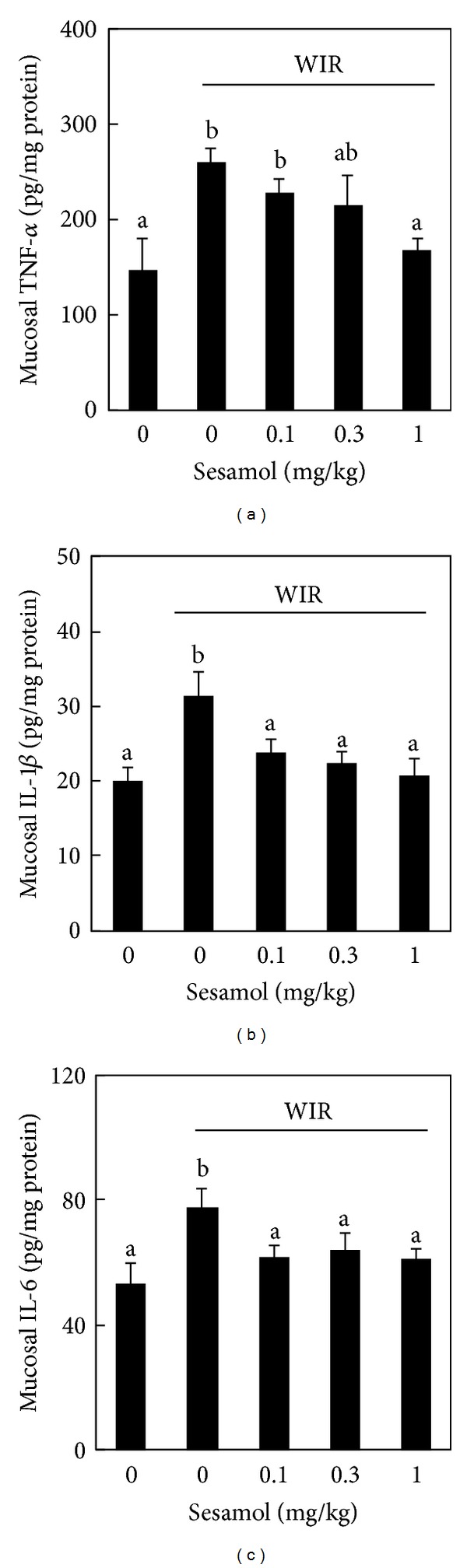
Effects of sesamol on mucosal pro-inflammatory cytokines production in water immersion restraint- (WIR-) treated rats. Rats were divided into five groups of five: Group I, non-WIR (normal) group; Group II, WIR (control) group rats were subjected to WIR; and Groups III–V, sesamol-plus-WIR group rats were orally given sesamol (0.1, 0.3, and 1 mg/kg, resp.) 30 min before WIR. Mucosal TNF-*α* (a), IL-1*β* (b), and IL-6 (c) levels were measured after WIR. Data are means ± standard deviation (SD). Significant differences between measurement traits were analyzed using one-way ANOVA. Different letters between groups indicate statistically significant differences (*P* < 0.05).

To examine the involvement of inflammation in sesamol-associated gastric mucosal protection against stress, mucosal TNF-*α*, IL-1*β*, and IL-6 were determined. WIR increased mucosal TNF-*α* (Figure 4(a)), IL-1*β* (Figure 4(b)), and IL-6 (Figure 4(c)) levels in the WIR group compared with those in the non-WIR group. Sesamol at the dose of 1 mg/kg significantly decreased mucosal TNF-*α* level in the sesamol-plus-WIR group compared with that in the WIR control group (Figure 4(a)). In addition, sesamol (sesamol-plus-WIR group) at the doses of 0.1, 0.3, and 1 mg/kg showed significant inhibition in the mucosal IL-1*β* (Figure 4(b)) and IL-6 (Figure 4(c)) production compared with those in the WIR control group.

### 3.5. Effects of Sesamol on NF-*κ*B Activation in WIR-Treated Rats

**Figure 5:**
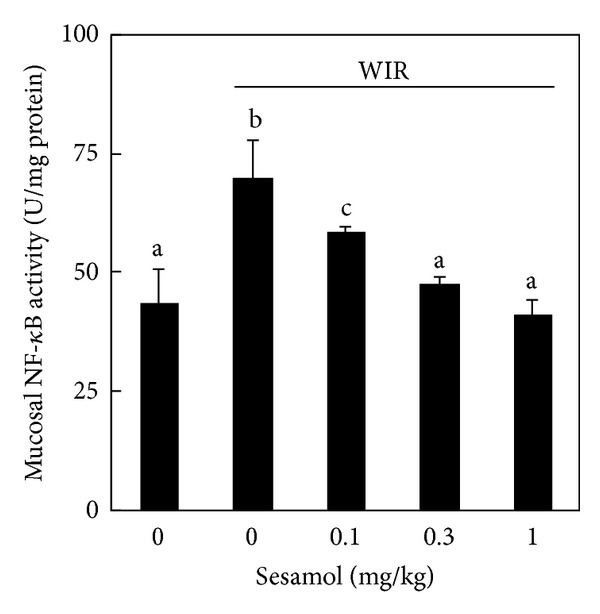
Effects of sesamol on nuclear factor (NF)-*κ*B activation in water immersion restraint- (WIR-) treated rats. Rats were divided into five groups of five: Group I, non-WIR (normal) group; Group II, WIR (control) group rats were subjected to WIR; and Groups III–V, sesamol-plus-WIR group rats were orally given sesamol (0.1, 0.3, and 1 mg/kg, resp.) 30 min before WIR. Mucosal NF-*κ*B activation was assessed after WIR. Data are means ± standard deviation (SD). Significant differences between measurement traits were analyzed using one-way ANOVA. Different letters between groups indicate statistically significant differences (*P* < 0.05).

To further investigate the anti-inflammatory effect of sesamol on stress-associated gastric inflammation, mucosal transcription factor NF-*κ*B activation was assessed. WIR increased mucosal NF-*κ*B activation in the WIR control group compared with that in the non-WIR group. Sesamol (sesamol-plus-WIR group) significantly inhibited mucosal NF-*κ*B activation compared with that in WIR control group (Figure 5).

### 3.6. Effects of Sesamol on Neutrophil Infiltration in WIR-Treated Rats

**Figure 6:**
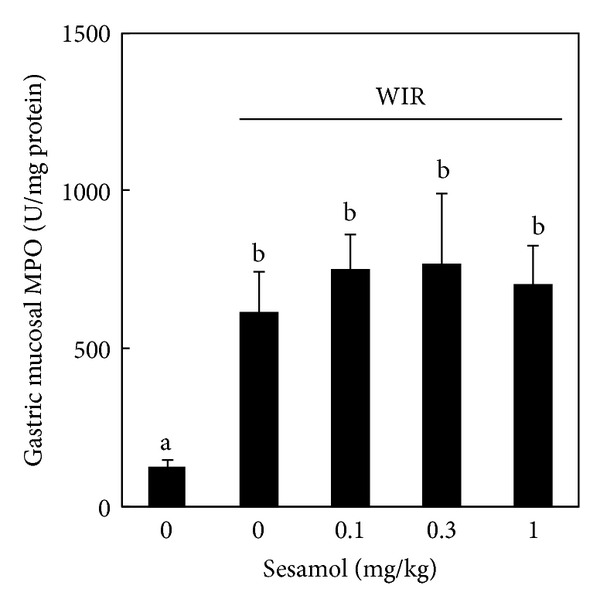
Effects of sesamol on neutrophil infiltration in water immersion restraint- (WIR-) treated rats. Rats were divided into five groups of five: Group I, non-WIR (normal) group; Group II, WIR (control) group rats were subjected to WIR; Groups III–V, sesamol-plus-WIR group rats were orally given sesamol (0.1, 0.3, and 1 mg/kg, resp.) 30 min before WIR. Mucosal myeloperoxidase (MPO) activity was assessed after WIR. Data are means ± standard deviation (SD). Significant differences between measurement traits were analyzed using one-way ANOVA. Different letters between groups indicate statistically significant differences (*P* < 0.05).

To investigate the role of neutrophil in the protective effect of sesamol on stress-associated gastric inflammation, gastric mucosal neutrophil infiltration was assessed by determining mucosal MPO activity. WIR increased mucosal MPO activity in the WIR control group compared with that in the non-WIR group. However, sesamol (sesamol-plus-WIR group) failed to affect the mucosal MPO activity compared with that in WIR control group (Figure 6).

### 3.7. Effects of Sesamol on Macrophage Infiltration in WIR-Treated Rats

**Figure 7:**
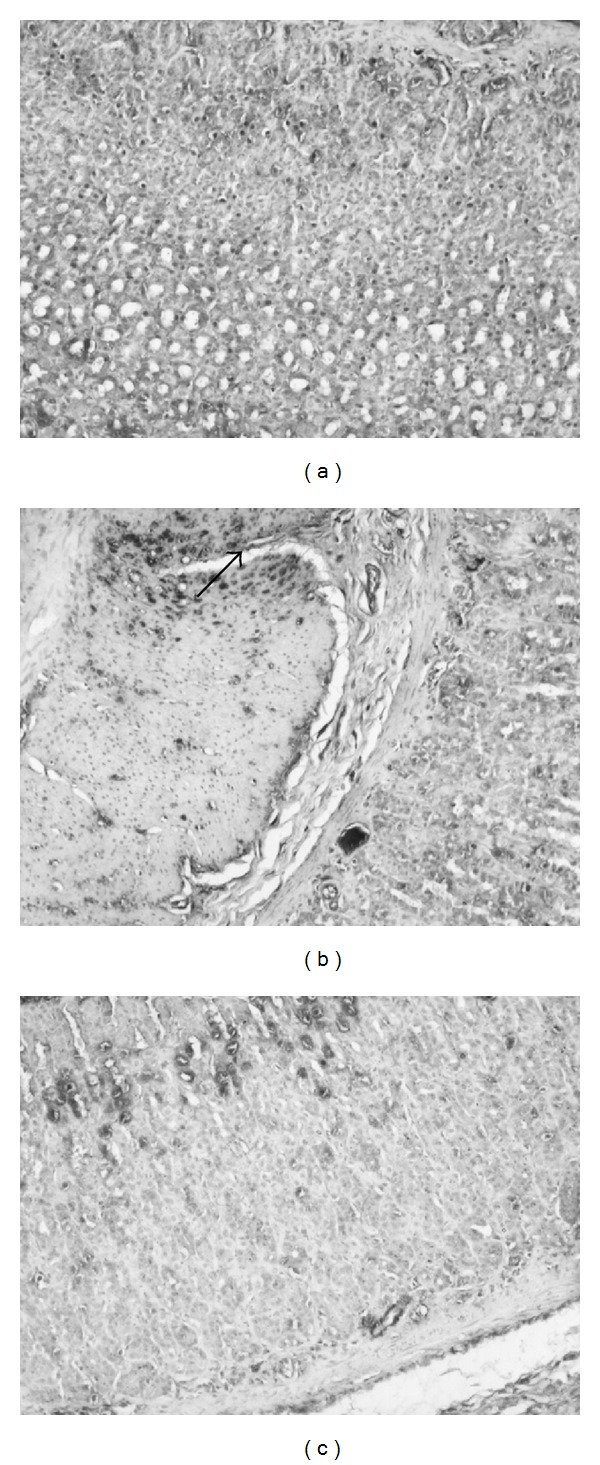
Effects of sesamol on CD68 expression in water immersion restraint- (WIR-) treated rats. Rats were divided into three groups: Group I, non-WIR (normal) group (a); Group II, WIR (control) group rats were subjected to WIR (b); and Group III, sesamol-plus-WIR group rats were orally given sesamol (1 mg/kg) 30 min before WIR (c). Photographs were taken at 100x. Arrow indicates intense staining of CD68 in submucosa.

The normal mucosa with basal level expression of CD68 was found in non-WIR group (Figure 7(a)). We found intense staining pattern for CD68 in WIR group in all muscularis, submucosa (muscularis mucosa), and mucosa layers of stomach, particularly in muscularis mucosa and vascular system (Figure 7(b)). Sesamol significantly decreased the staining of CD68, particularly in muscularis mucosa area in mucosal area in sesamol-plus-WIR group compared with that in WIR control group (Figure 7(c)).

## 4. Discussion

Sesamol potently attenuated gastric mucosal injury induced by WIR in rats. Sesamol decreased stress-induced gastric mucosal hemorrhage and ulceration, but it affected neither oxidative stress nor mucus secretion in gastric mucosa. Sesamol decreased pro-inflammatory cytokine production and NF-*κ*B activation. Further, sesamol decreased macrophage but not neutrophil infiltration into gastric mucosa in WIR-treated rats. We suggest that sesamol protects against stress-induced gastric damage by decreasing macrophage activation and gastric inflammation in rats.

 Lipid peroxidation and mucus deficiency may not be involved in the protective effect of sesamol against stress-induced gastric mucosal injury. Lipid peroxidation and mucus deficiency have been reported to be involved in the physiopathogenesis of various gastric mucosal injuries [22–25]. However, both LPO and mucus levels were not altered by WIR or sesamol in our model. In the present study, oxidative stress might not play an important role in sesamol's protection against WIR-induced gastric hemorrhage and ulceration.

 Sesamol may attenuate stress ulcer by inhibiting mucosa inflammation. Gastric mucosal inflammation is involved in the pathogenesis of various gastric mucosal injuries [26]. The cytokine network may thus participate in inflammatory responses to various ulcerogenic factors, resulting in enhancement of inflammation and injury in gastric mucosa [12]. Overproduction of inflammatory cytokine, such as TNF-*α*, is responsible for stress-induced gastric mucosal injury [11] and other types of gastric injury [12]. In addition, pretreatment of anti-TNF-*α* antibody prevented stress-induced gastric injury by decreasing TNF-*α* expression in rat gastric mucosa in WIR model [11]. In addition, the transcription factor NF-*κ*B activation is involved in acute injury of stomach. The activation of NF-*κ*B induces gene programs leading to transcription of pro-inflammatory mediators that promote inflammation [19]. Sesamol attenuated the recruitment of inflammatory cells, mast cells, CD68(+) Kupffer cells, and neutrophils in liver injury [27]. Reducing the recruitment of inflammatory cells in the gastric mucosa by sesamol might attenuate cytokine production [13]. In addition, sesamol inhibits the NF-*κ*B activation during systemic inflammation [13], and thus it reduces the transcription of pro-inflammatory cytokines that induces inflammation and gastric ulcer. In the present study, sesamol significantly decreased mucosal cytokine production and NF-*κ*B expression. It is likely that sesamol attenuates stress-related gastric mucosal ulceration and hemorrhage by inhibiting NF-*κ*B expression and inflammation.

 Inhibiting macrophages activation may play an important role in sesamol-associated gastric mucosal protection against stress. During gastric mucosal injury, neutrophil and macrophage infiltrate into inflammatory area [28–30], both of which are involved in the generation of pro-inflammatory cytokines and gastric mucosal damage [31–33]. However, in our model, sesamol decreased the infiltration of macrophage but not neutrophil. It seems that inhibiting the activation of macrophage but not neutrophil is associated with the protective effect of sesamol against acute gastric mucosal injury induced by WIR.

## 5. Conclusion

Taken together, sesamol's protective effect against mucosal injury in WIR might be via attenuations of cytokines and NF-*κ*B activation in rats. However, the exact mechanism involved in the sesamol-related gastric mucosal protection requires further studies. Further, although sesamol showed promising gastric protective effects against WIR-induced mucosal injury in the animal study, to increase its potential for clinical use, more clinically oriented investigations are needed.
